# Allopregnanolone mediates the exacerbation of Tourette-like responses by acute stress in mouse models

**DOI:** 10.1038/s41598-017-03649-1

**Published:** 2017-06-13

**Authors:** Laura J. Mosher, Sean C. Godar, Marianela Nelson, Stephen C. Fowler, Graziano Pinna, Marco Bortolato

**Affiliations:** 1 Dept. of Pharmacology and Toxicology, College of Pharmacy; University of Utah, Salt Lake City (UT), USA; 2 Dept. of Pharmacology and Toxicology, School of Pharmacy, University of Kansas, Lawrence, KS USA; 3 The Psychiatric Institute, Dept. of Psychiatry, College of Medicine, University of Illinois at Chicago, Chicago, IL USA; 4 Tourette Syndrome Center, University of Cagliari, Cagliari, Italy

## Abstract

Tourette syndrome (TS) is a neuropsychiatric disorder characterized by multiple tics and sensorimotor abnormalities, the severity of which is typically increased by stress. The neurobiological underpinnings of this exacerbation, however, remain elusive. We recently reported that spatial confinement (SC), a moderate environmental stressor, increases tic-like responses and elicits TS-like sensorimotor gating deficits in the D1CT-7 mouse, one of the best-validated models of TS. Here, we hypothesized that these adverse effects may be mediated by neurosteroids, given their well-documented role in stress-response orchestration. Indeed, SC increased the levels of progesterone, as well as its derivatives 5α-dihydroprogesterone and allopregnanolone, in the prefrontal cortex (PFC) of D1CT-7 mice. Among these steroids, however, only allopregnanolone (5–15 mg/kg, IP) dose-dependently exacerbated TS-like manifestations in D1CT-7, but not wild-type littermates; these effects were countered by the benchmark anti-tic therapy haloperidol (0.3 mg/kg, IP). Furthermore, the phenotypic effects of spatial confinement in D1CT-7 mice were suppressed by finasteride (25–50 mg/kg, IP), an inhibitor of the main rate-limiting enzyme in allopregnanolone synthesis. These findings collectively suggest that stress may exacerbate TS symptoms by promoting allopregnanolone synthesis in the PFC, and corroborate previous clinical results pointing to finasteride as a novel therapeutic avenue to curb symptom fluctuations in TS.

## Introduction

Tourette syndrome (TS) is a neuropsychiatric condition characterized by multiple, recurring motor and phonic tics^1^. These movements and utterances are typically executed in response to *premonitory sensory phenomena*, defined as uncomfortable feelings of fixation on intrusive somatic cues^2, 3^. Premonitory sensory phenomena are associated with dysfunctions of sensory gating^4, 5^, the perceptual domain that filters out irrelevant or redundant information; accordingly, TS patients exhibit deficits in the prepulse inhibition of the startle reflex (PPI)^6, 7^, one of the best-characterized cross-species operational indices of gating integrity.

Tics are highly variable in intensity and frequency. Among other factors, these symptom fluctuations are posited to reflect the impact of select physical and psychological stressors^2, 8, 9^. The neurobiological mechanisms whereby tics are exacerbated by contextual triggers, however, are poorly understood; as a result, no pharmacological interventions are currently available to prevent or mitigate tic aggravation in response to stress.

Animal models of tic disorders are essential tools for testing of biological hypotheses on tic ontogeny and/or developing novel therapies^10^. One of the best-characterized animal models of TS is the D1CT-7 mouse, a transgenic mutant generated by the attachment of the D1 dopamine receptor promoter to the gene encoding the neuro-potentiating cholera toxin. These animals display spontaneous tic-like clonic jerks that are reduced by benchmark therapies for TS, such as the antipsychotic haloperidol^11, 12^. We recently documented that D1CT-7 mice display exacerbated tic-like responses and PPI deficits in response to a naturalistic environmental stressor, consisting of a 20-min spatial confinement (SC) within a 10-cm diameter cylindrical enclosure in their home cages^13^. Notably, both these behavioral responses were fully suppressed by haloperidol^13^.

Neuroactive steroids, such as the neurosteroid allopregnanolone (3α,5α, tetrahydroprogesterone; AP), exert a critical influence in the modulation of the allostatic response to stress. AP acts as a positive allosteric modulator of GABA-A receptors^14^, and this mechanism is posited to promote resilience and mitigate the adverse neurobehavioral effects of short-term stress^15, 16^. Furthermore, the behavioral effects of AP are contributed by other receptors, including pregnane X^17^ and progesterone receptors^18^. The rate-limiting step in AP synthesis is catalyzed by the enzyme 5α-reductase, which saturates the 4,5-double bond of the A ring of progesterone. The product of this chemical reaction, 5α-dihydroprogesterone (DHP), is then converted to AP by 3α-hydroxysteroid dehydrogenase^19, 20^. Notably, we previously showed that the 5α-reductase inhibitor finasteride elicited therapeutic properties in TS patients^21, 22^ and increased PPI deficits in rodents treated with dopaminergic agonists^23–25^.

Based on this background, we designed the present study to test the implication of neurosteroids in the effects of SC on tic-like responses and PPI deficits in D1CT-7 mice. Our analyses focused particularly on the prefrontal cortex (PFC), given that rich evidence has established that this region is particularly sensitive to the effects of acute stress on both neurosteroid synthesis^26^ and plays a fundamental role in the control of tics (and its modulation by stress) in TS^27, 28^.

## Results

### SC increases neuroactive steroid levels in the prefrontal cortex (PFC)

**Figure 1:**
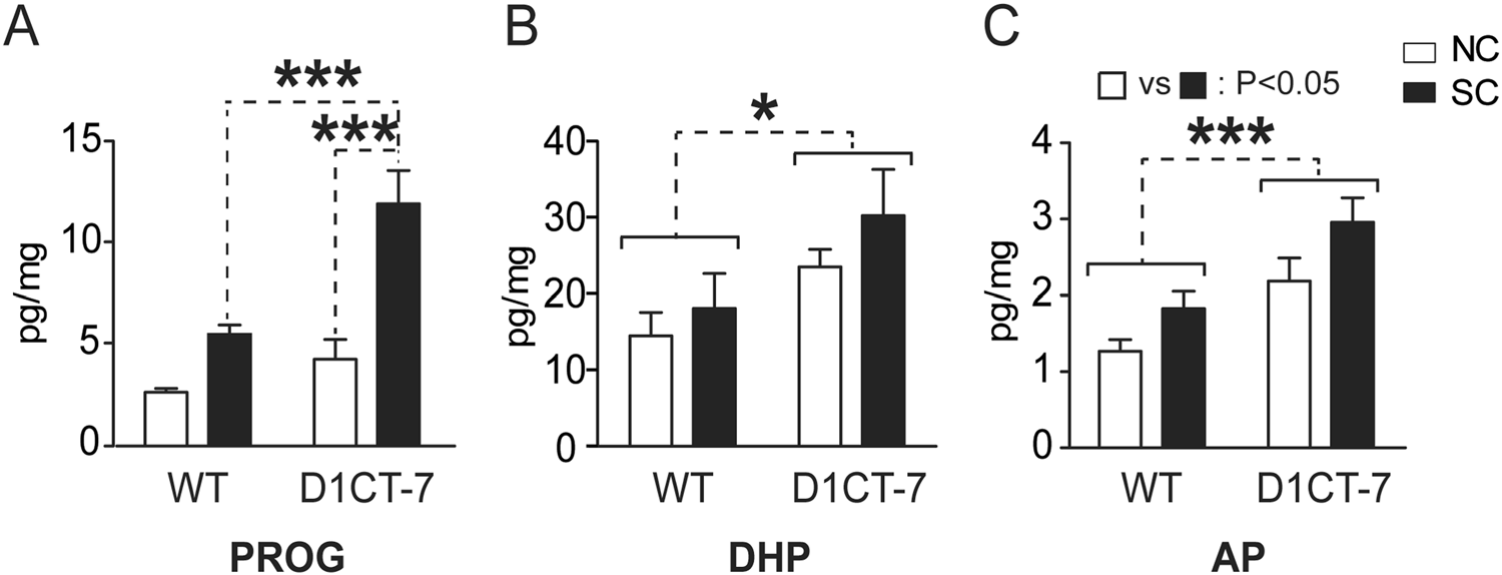
Levels of (**A**) progesterone, (**B**) 5α-dihydroprogesterone (DHP) and (**C**) allopregnanolone (AP) in the prefrontal cortex of D1CT-7 and wild-type (WT) littermates following spatial confinement (SC). All measurements were performed after 20 min of SC. Data are shown as means ± SEM. *P < 0.05, and ***P < 0.001 for comparisons indicated by dashed lines. Main effects for genotype are indicated as comparisons between brackets. Main effects for SC are indicated as comparison between symbols. N = 4–7/group. NC, no spatial confinement. For further details, see text.

The first study was aimed at the measurement of steroid levels in the PFC of D1CT-7 and wild-type (WT) littermates following SC (Fig. 1). The analysis of progesterone content (Fig. 1A) revealed a significant genotype × confinement interaction [F(1,17) = 8.15, *P* < 0.01], reflecting significant differences between confined D1CT-7 mice and either WT controls (*P* < 0.001) or non-confined D1CT-7 mice (*P* < 0.001; Newman-Keuls). In contrast, DHP levels (Fig. 1B) were higher in D1CT-7 mice than WT controls [Main effect for genotype: F(1,21) = 6.03, *P* < 0.05]; however, SC had no significant effect on the content of this neurosteroid. Furthermore, no significant interactions between genotype and stress were found. The PFC concentrations of AP (Fig. 1C) in D1CT-7 mice were higher than those detected in their WT littermates [F(1,22) = 14.97, *P* < 0.001]. The same neurosteroid was found to be enhanced by SC [F(1,22) = 6.38, *P* < 0.05]. No significant genotype × stress interaction was detected. Finally, no significant differences were found in the content of pregnanolone, the 3α,5β-reduced derivative of progesterone (Table S1).

### AP selectively increases tic-like responses and induces PPI deficits in D1CT-7, but not WT mice

**Figure 2:**
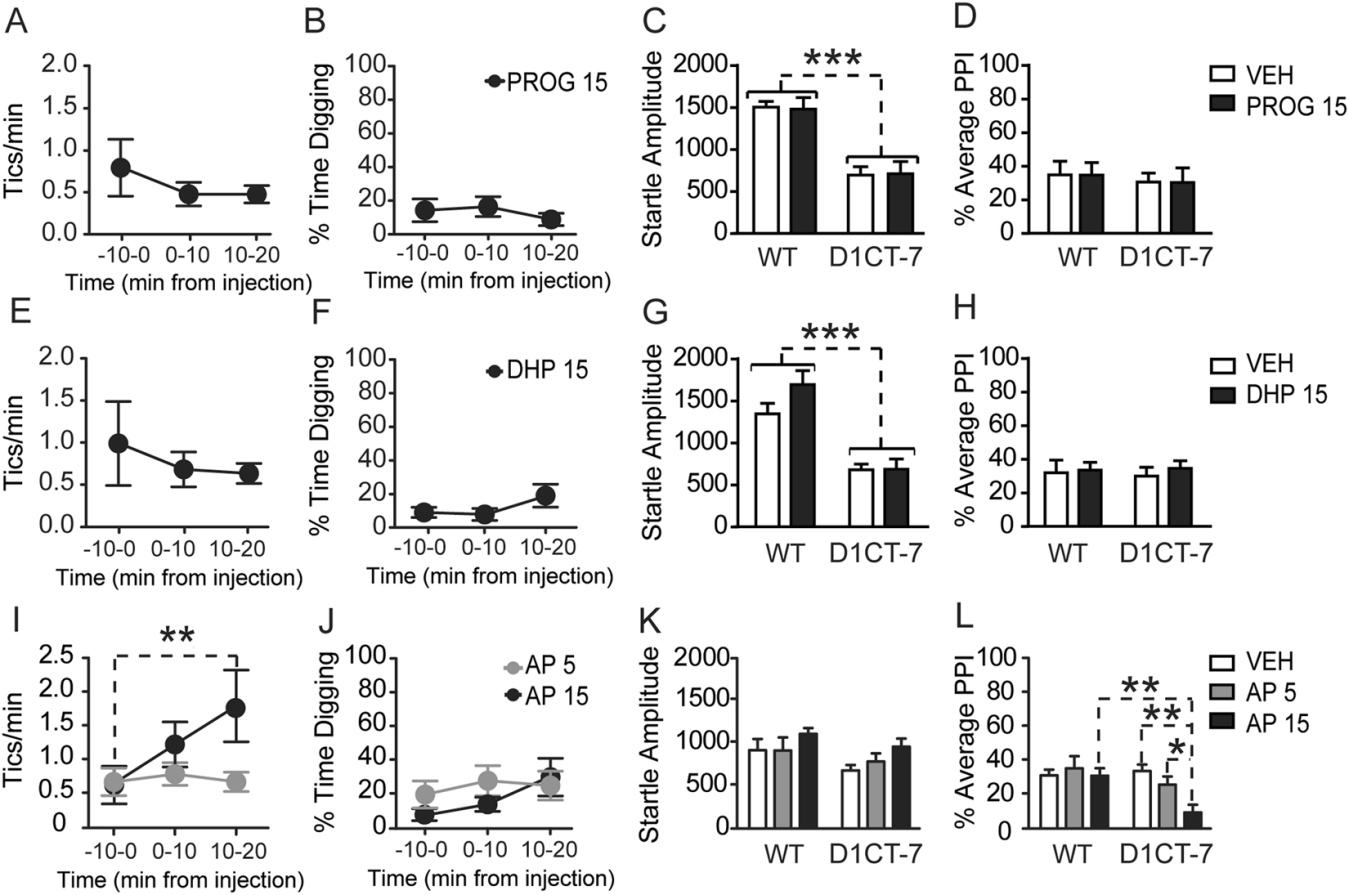
Effects of (**A–D**) progesterone (PROG), (**E–H**) 5α-dihydroprogesterone (DHP) and (**I–L**) allopregnanolone (AP) on behavioral phenotypes related to Tourette syndrome. Data are shown as means ± SEM. *P < 0.05, **P < 0.01 and ***P < 0.001 for comparisons indicated by dashed lines. Main effects for genotype are indicated as comparisons between brackets. Doses are indicated in mg/kg (IP). N = 8–13/group. VEH, vehicle; WT, wild-type; PPI, prepulse inhibition of the startle. For further details, see text.

Given that our previous results showed that SC was associated with increased levels of progesterone, DHP and AP, we next tested whether the administration of any of these steroids may reproduce the adverse effects of this stressor on tic-like responses and PPI deficits^13^. Behavioral testing began 10 min after steroid injections. Neither progesterone (15 mg/kg, IP) (Fig. 2A–D) nor DHP (15 mg/kg, IP) (Fig. 2E–H) elicited any significant behavioral change in D1CT-7 mice. The only significant effect detected by these analyses was a significant reduction in startle amplitude in D1CT-7, in comparison with WT counterparts [Fig. 2C: F(1,28) = 45.65, *P* < 0.001; Fig. 2G: F(1,28) = 44.92, *P* < 0.001], as previously reported^13^. However, this effect was not modified by any treatment.

In contrast with these findings, AP (5–15 mg/kg, IP) significantly increased tic-like responses in D1CT-7 mice (Fig. 2I) [F(2,30) = 4.27, *P* < 0.05; 2-way ANOVA]. Specifically, the 15 mg/kg dose significantly increased the frequency of tic-like responses between 10 and 20 min after the injection (*P* < 0.01; Newman-Keuls) (Fig. 2I). Conversely, AP did not affect digging behavior in either genotype (Fig. 2J). The analysis of startle magnitude (Fig. 2K) showed only statistical trends with respect to main effects of genotype [F(1,57) = 3.62, *P* = 0.06] and AP treatment [F(2,57) = 2.53, *P* = 0.09]; however, no significant interactions between these two factors were detected. Notably, a significant genotype × treatment interaction was found for the mean PPI values [F(2.57) = 3.25, *P* < 0.05] (Fig. 2L). Post-hoc analyses showed that the 15 mg/kg dose of AP induced a significant reduction in mean PPI values as compared with WT counterparts treated with the same dose (*P* < 0.01), as well as D1CT-7 treated with either vehicle (*P* < 0.01) or 5 mg/kg of AP (*P* < 0.05).

### D1CT-7 mice do not exhibit alterations in the levels of key neurosteroid-binding GABA-A receptor subunits in the PFC

To explore whether the observed effects of AP may reflect changes in GABA-A receptors, we measured the levels of key GABA-A subunits that are posited to modulate the activity of AP and other neuroactive steroids, namely α1, α4, δ and π. Nevertheless, the expression of these proteins in the PFC (as captured by western blotting) was equivalent in WT and D1CT-7 littermates (Fig. S1), indicating that the actions of AP were not supported by apparent changes in GABA-A stoichiometry.

### AP increases locomotor activity in D1CT-7, but not WT mice

**Figure 3:**
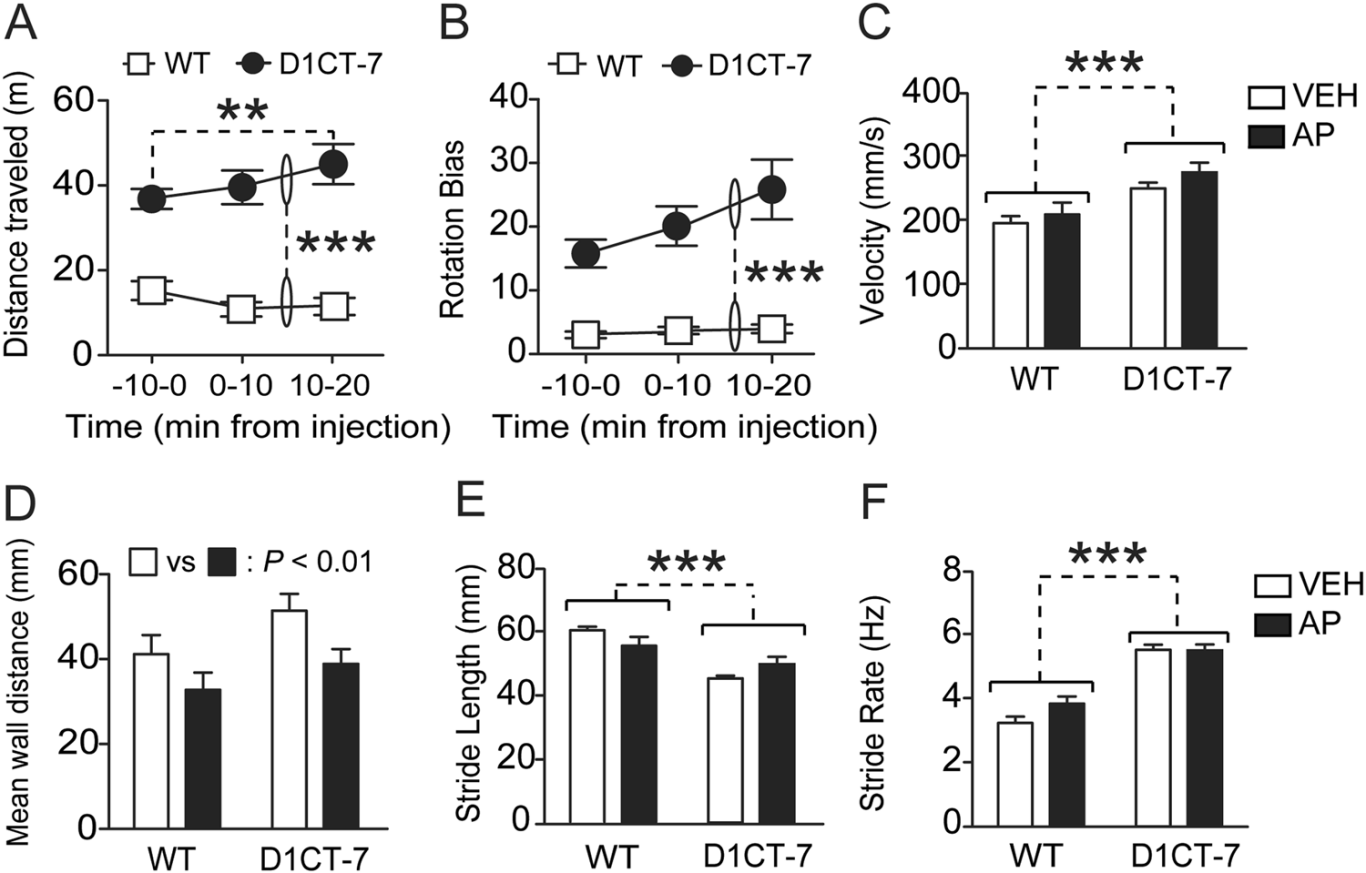
Effects of allopregnanolone (AP; 15 mg/kg, IP) on the locomotor activity of D1CT-7 and wild-type (WT) mice. Data are shown as means ± SEM. **P < 0.01 and ***P < 0.001 for comparisons indicated by dashed lines. Main effects for genotype are indicated as comparisons between brackets. Doses are indicated in mg/kg (IP). N = 12–13/group. VEH, vehicle. For further details, see text.

Next, we examined whether the behavioral changes induced by 15 mg/kg of AP were accompanied by variations in locomotor activity. As previously observed^10^, D1CT-7 mice exhibited significantly higher locomotion [Main effect of genotype: F(1,23) = 58.42, *P* < 0.001]. In addition, a significant genotype × treatment interaction [F(2,46) = 5.34, *P* < 0.01] revealed that AP selectively increased the locomotor activity between 10 and 20 min after injection in D1CT-7 mice (*P* < 0.01 in comparison with baseline), but not WT controls (Fig. 3A). The analysis of rotation bias (Fig. 3B) and velocity (Fig. 3C) revealed that these indices were significantly elevated in D1CT-7 mice [Main genotype effects: Rotation bias: F(1,23) = 19.91, *P* < 0.001; Velocity: F(1,18) = 2.39, *P* < 0.001], but were not altered by AP administration in either genotype. Conversely, D1CT-7 and WT mice showed similar thigmotactic behavior (Fig. 3D), but AP increased the average distance from the walls of the arena in both genotypes [Main effect: F(1,19) = 10.08, *P* < 0.01]. In confirmation of previous data^29^, D1CT-7 mice displayed a decreased stride length [F(1,18) = 22.81, *P* < 0.001] (Fig. 3E) and an increased stride rate [F(1,18) = 82.33, *P* < 0.001] (Fig. 3F) compared to WT mice. Neither parameter, however, was affected by AP treatment.

### Haloperidol countered the enhancement in tic-like responses induced by AP

**Figure 4:**
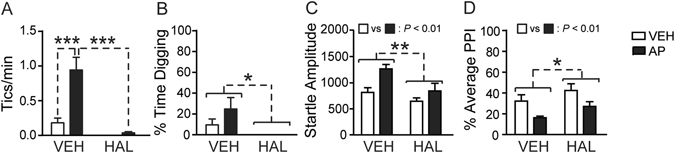
Combined effects of haloperidol (HAL; 0.3 mg/kg, IP) and allopregnanolone (AP; 15 mg/kg, IP) on behavioral phenotypes related to Tourette syndrome in D1CT-7 mice. Data are shown as means ± SEM. **P < 0.01 and ***P < 0.001 for comparisons indicated by dashed lines. N = 8–9/group. VEH, vehicle; PPI, prepulse inhibition. For further details, see text.

We previously documented that haloperidol (0.3 mg/kg, IP) suppressed the increase in tic-like jerks and PPI deficits induced by SC in D1CT-7 mice. Given that our previous resulted showed that AP treatment led to similar effects as those caused by SC, we tested whether its effects may be countered by haloperidol. D1CT-7 mice were pretreated with haloperidol (0.3 mg/kg, IP) 20 min prior to AP (15 mg/kg, IP) administration. Analysis of tic-like behaviors (Fig. 4A) revealed a significant haloperidol × AP interaction [F(1,31) = 12.92, *P* < 0.001]. This effect indicated that, while AP increased tic-like responses, pre-treatment with haloperidol suppressed this response (Ps < 0.001). Conversely, digging (Fig. 4B) was suppressed by haloperidol, irrespective of AP administration [Main effect of haloperidol: F(1,31) = 7.02, *P* < 0.05]. Startle analysis (Fig. 4C) revealed that haloperidol reduced the mean amplitude of this parameter [Main effect of haloperidol: F(1,31) = 8.63, *P* < 0.01], while AP increased it [Main effect of AP: F(1,31) = 10.38, *P* < 0.01]; however, no significant interaction between these two treatments was detected. As expected, PPI was reduced by AP [Main effect of AP: F(1,31) = 9.58, *P* < 0.01] and increased by haloperidol [Main effect of haloperidol: F(1,31) = 4.41, *P* < 0.05], but these two effects did not significantly interact (Fig. 4D).

### Finasteride counters the behavioral and neuroendocrine effects of SC

**Figure 5:**
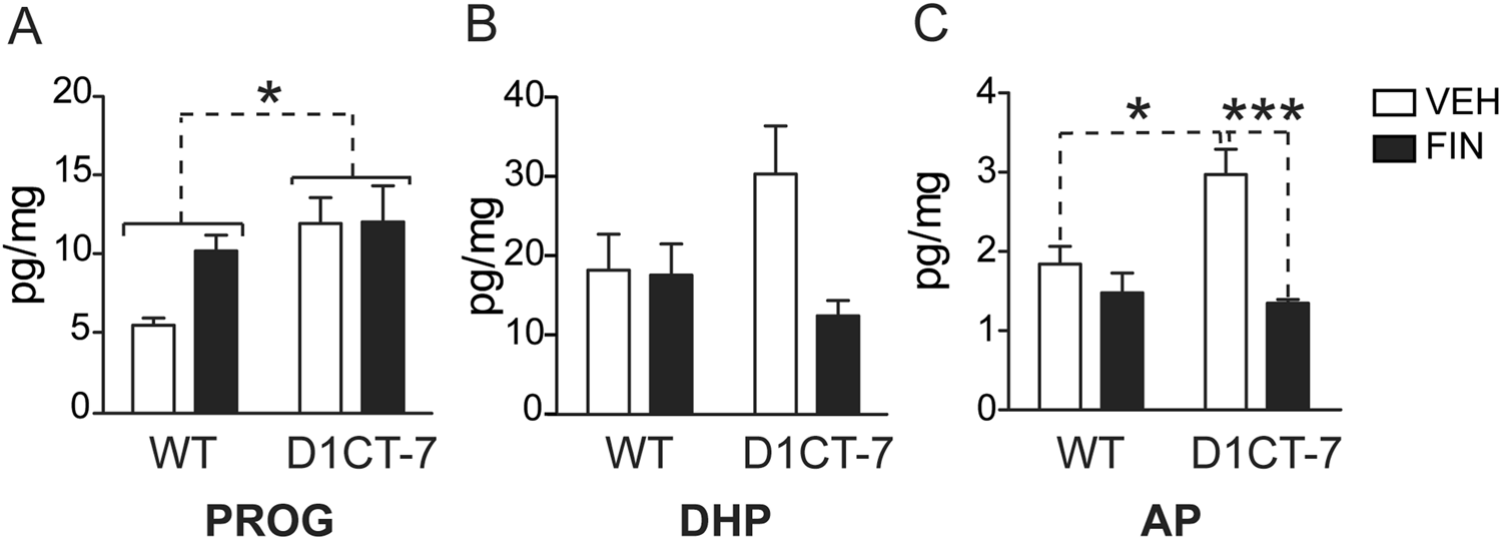
Effects of finasteride (FIN; 50 mg/kg, IP) on levels of (**A**) progesterone, (**B**) 5α-dihydroprogesterone (DHP) and (**C**) allopregnanolone (AP) in the prefrontal cortex of D1CT-7 and wild-type (WT) littermates following spatial confinement (SC). Data are shown as means ± SEM. **P < 0.01, and ***P < 0.001 for comparisons indicated by dashed lines. Main effects for genotype are indicated as comparisons between brackets. Main effects for SC are indicated as comparison between symbols. N = 5–7/group. VEH, vehicle. For further details, see text.

To further assess whether AP mediated the effects of SC in D1CT-7 mice, we then verified whether the observed increase in tics and PPI deficits induced by SC could be countered by finasteride. While progesterone levels in the PFC of confined D1CT-7 mice were confirmed to be higher than those in wild type controls [Main effect of genotype: F(1,20) = 7.51, *P* < 0.05], the content of this steroid was not affected by finasteride treatment (Fig. 5A). The analysis of finasteride’s effects on DHP revealed a statistical trend for a reduction in DHP levels in both wild type and D1CT-7 mice [F(1,20) = 4.05, *P* = 0.06] (Fig. 5B). Finally, ANOVA detected a significant genotype × treatment interaction on AP levels [F(1,22) = 6.77, *P* < 0.05]. Post-hoc analyses revealed that finasteride fully countered (*P* < 0.001) the enhancement in AP produced by SC in D1CT-7 mice (*P* < 0.05) (Fig. 5C).

**Figure 6:**
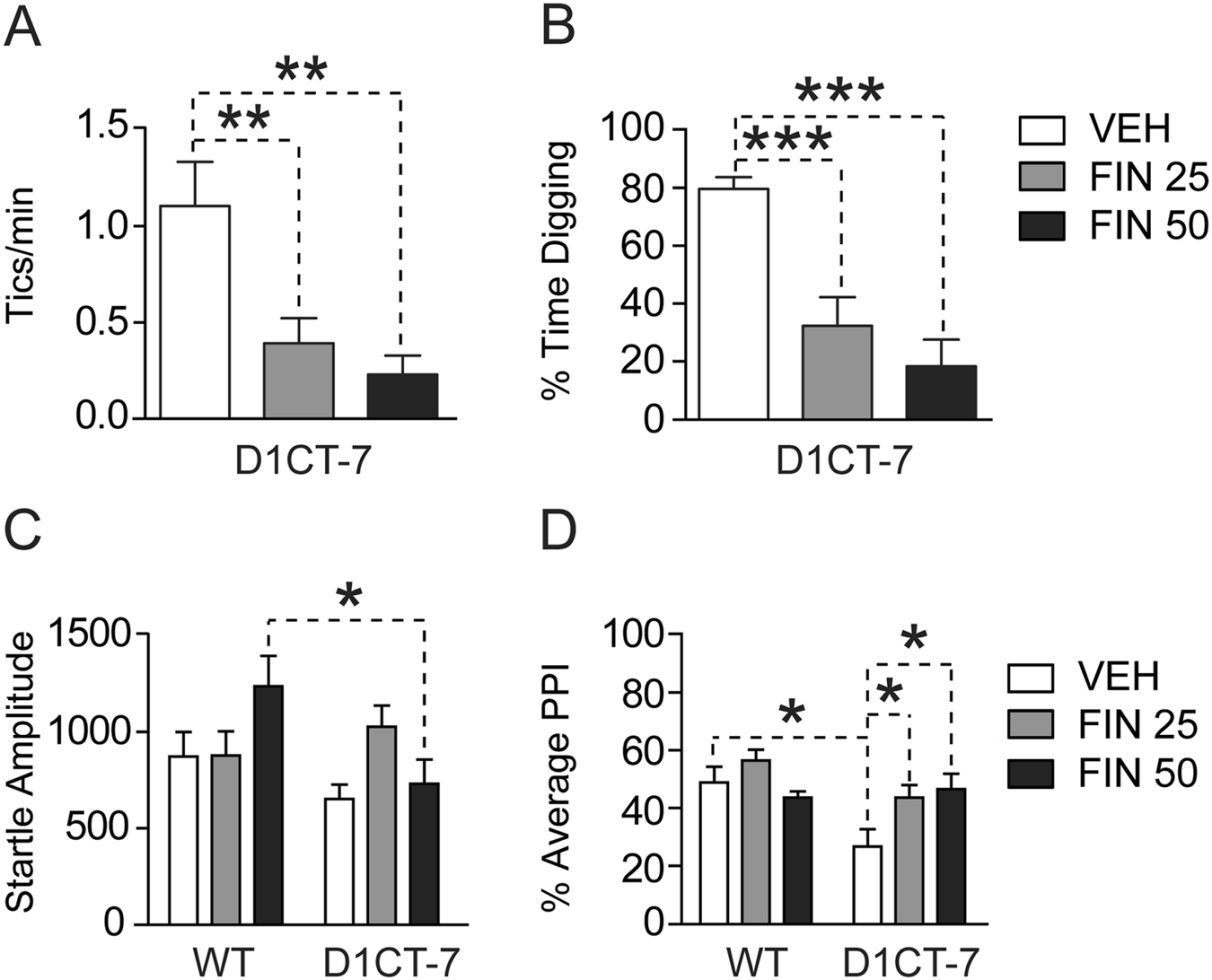
Effects of finasteride (FIN; 25–50 mg/kg, IP) on behavioral phenotypes related to Tourette syndrome. Data are shown as means ± SEM. *P < 0.05, **P < 0.01 and ***P < 0.001 for comparisons indicated by dashed lines. Doses are indicated in mg/kg (IP). N = 8/group. VEH, vehicle; WT, wild-type; PPI, prepulse inhibition of the startle. For further details, see text.

We next tested whether the reduction in AP levels induced by finasteride could be paralleled by the normalization of behavioral responses in spatially-confined D1CT-7 mice (Fig. 6). Finasteride dose-dependently reduced the tic-like bursts (Fig. 6A) induced by SC in D1CT-7 mice [F(2,21) = 8.15, *P* < 0.01; *P* < 0.01 for comparisons between vehicle and 25 mg/kg of finasteride; *P* < 0.01 for comparisons between vehicle and 50 mg/kg of finasteride]. Finasteride also reduced digging behavior (Fig. 6B) in D1CT-7 mice [F(2,21) = 15.51, *P* < 0.01; *P* < 0.001 for comparisons between vehicle and 25 mg/kg of finasteride; *P* < 0.001 for comparisons between vehicle and 50 mg/kg of finasteride]. The analysis of startle amplitude revealed a significant genotype × treatment interaction [F(2,42) = 3.56, *P* < 0.05], which reflected a greater startle-reducing effect of finasteride (50 mg/kg) in D1CT-7 than WT mice (*P* < 0.05) (Fig. 6C). PPI analyses (Fig. 6D) also revealed significant genotype × treatment interactions [F(2,42) = 3.63, *P* < 0.05]; *post hoc* scrutiny of this effect confirmed that SC significantly reduced PPI in vehicle-treated D1CT-7 mice (*P* < 0.05). In addition, finasteride ablated the gating deficits induced by SC in D1CT-7 mice at both the 25 mg/kg (*P* < 0.05) and 50 mg/kg (*P* < 0.05) doses.

Finally, the analysis of locomotor activity in both WT and D1CT-7 mice (Fig. S2) showed a significant genotype × treatment × time interaction [F(10,210) = 2.37, *P* < 0.05]. Overall, while D1CT-7 mice exhibited higher locomotor activity than their WT counterparts, the 50 mg/kg dose of finasteride exerted a profound hypolocomotive effect in both genotypes.

### AP opposes the effect of finasteride on gating deficits, but not tic-like responses, in D1CT-7 mice

**Figure 7:**
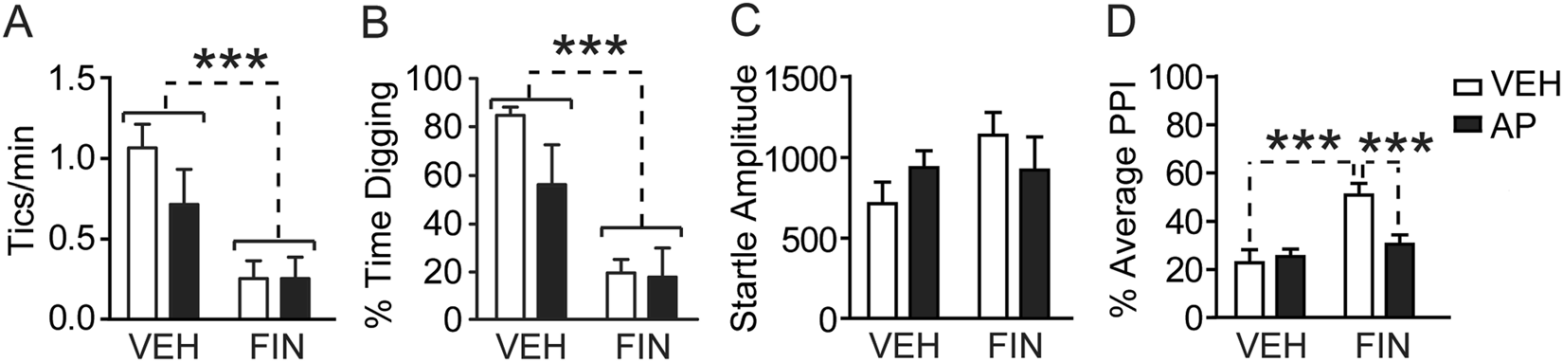
Combined effects of finasteride (FIN; 25 mg/kg, IP) and allopregnanolone (AP; 15 mg/kg, IP) on behavioral phenotypes related to Tourette syndrome in D1CT-7 mice. Data are shown as means ± SEM. ***P < 0.001 for comparisons indicated by dashed lines. Main effects for genotype are indicated as comparisons between brackets. N = 8/group. Abbreviations: VEH, vehicle; PPI, prepulse inhibition. For further details, see text.

Lastly, we verified whether AP may reverse the ability of finasteride to attenuate the effects of SC in D1CT-7 mice. Finasteride (25 mg/kg, IP) and AP (15 mg/kg, IP) were administered at 40 and 10 min prior to space confinement, respectively. Only the 25 mg/kg dose of finasteride was used for these studies, since initial observations showed that the combination of AP with the higher dose of finasteride had profound sedative effects in D1CT-7 mice. Finasteride was confirmed to reduce tic-like behaviors [Main effect: F(1,28) = 16.23, *P* < 0.001] (Fig. 7A) and digging responses [F(1,28) = 23.21 *P* < 0.001] (Fig. 7B); however, these effects were not countered by AP injection. In contrast with these findings, analysis of PPI revealed a significant finasteride × AP interaction [F(1,28) = 7.10, *P* < 0.05], which reflected the ability of AP to reverse (*P* < 0.001) the ameliorating effects of finasteride (*P* < 0.001) on this endophenotype (Fig. 7D).

## Discussion

The present results show that, in the D1CT-7 mouse model of TS,﻿﻿ acute stress exacerbates tic-like responses and impairs PPI by promoting the synthesis of the neurosteroid AP.﻿ Indeed, while these responses were associated with a generalized enhancement in progesterone, DHP and AP in the PFC, only the latter steroid elicited behavioral abnormalities akin to those observed following SC. Furthermore, the 5α-reductase inhibitor finasteride led to a normalization of stress-induced behavioral changes and of cortical AP levels in D1CT-7 mice, without eliciting any such effects in wild type littermates.

As mentioned above, tics are characterized by striking fluctuations in intensity and frequency, which are greatly contributed by the influence of select environmental triggers^8, 9^. Against this backdrop, the results of this study provide the first evidence of a mechanism that may be responsible for the adverse effects of stress on TS symptoms. The translational relevance of these results is underscored by our previous observations and open-label trials, documenting the therapeutic action of finasteride in adult male TS patients^21, 22, 30^. The present data are also in agreement with our finding that sleep deprivation, a common trigger for tic exacerbation^9, 31^, leads to sensorimotor gating deficits in rats via the enhancement of AP biosynthesis in the PFC^32^.

The observed increase in neurosteroid concentrations in the PFC is in alignment with their well-documented role in the regulation of stress responses^17^. Our studies did not explore the mechanisms whereby SC increases cortical progesterone levels. A possible explanation for this effect may be the upregulation of the translocator protein 18KDa (TSPO), which has been observed following acute stress in other rodent models^33^. TSPO transports cholesterol into the inner mitochondrial membrane, thereby enabling the conversion of cholesterol into pregnenolone, the direct precursor of progesterone^34, 35^.

Acute stress has been shown to increase 5α-reductase expression in the PFC^36^. Although the neurophysiological role of AP in humans in response to stress may be different from that recognized in rodents^37^, preliminary observations suggest that acute stress may also increase AP biosynthesis in humans^38^. The notion that the stress-induced increase in AP is aimed at reducing anxiety is of particular interest in the context of TS. Indeed, tic execution in TS patients is generally regarded as a response to stressful stimuli that mitigates the discomfort associated with premonitory urges^3^, and, indeed, tic severity has been found to be negatively correlated with cortisol levels^39^. This idea suggests that tic execution may be a by-product of the mechanisms of stress coping mounted by the cortex to offset the anxiogenic effects of stress itself^9^. Furthermore, tics are typically preceded by uncomfortable sensory phenomena^3, 40^, which often reflect a psychological fixation on specific somatic cues and are typically exacerbated by environmental stress^2^. Although sensory phenomena are subjective in nature and cannot be captured in animal models, the underlying alterations in information-processing have been related to deficits in PPI of the startle. Accordingly, a reduction of this index has been observed in TS patients^6, 7^ and may be a key phenotypic marker to assess construct validity in TS models^5, 10^. Previous work from our group has provided support for a role of neurosteroids in the regulation of PPI and finasteride pretreatment can prevent PPI deficits, which are induced by dopaminergic agonists as well as environmental stressors^23, 25, 32^. Furthermore, our work has shown that these effects can be regulated by the PFC, as well as by the nucleus accumbens, but not the dorsal striatum^24^.

The molecular mechanisms whereby AP leads to a robust exacerbation of tic-like behaviors and PPI deficits in D1CT-7 mice remain elusive. Although no alterations of cortical GABA-A receptor subunit composition were detected in this mouse model, it is conceivable that the behavioral outcomes of AP may be contributed by the positive allosteric modulation of GABA-A receptors in the PFC. Both GABA-A receptors and neurosteroidogenic enzymes are expressed in the same cortical pyramidal neurons^41^, providing a mechanism for excessive inhibition of glutamatergic neurons in the PFC; in turn, this process may result in the activation of striatum and other subcortical areas. Accordingly, stress has been shown to impair the function of the PFC^42^. In addition, the effects of AP may also be contributed by other mechanisms, such as the activation of membrane progesterone or pregnane X receptors. Alternatively, we cannot rule out that some of these actions may be due to the conversion of AP into its sulfo-conjugated derivative, AP sulfate, which acts as a negative allosteric modulator of NMDA glutamate receptors^43^. Irrespective of the specific receptors involved in the transduction of AP-mediated signals, exacerbation of Tourette-like manifestations may also be facilitated by AP-driven stimulation of dopamine release in the dorsal and ventral striatum^44^. In keeping with this concept, our data indicates that the dopamine receptor antagonist haloperidol reverses the enhancement in tics produced by AP, and countered - albeit not selectively – the PPI deficits induced by this neurosteroid in D1CT-7 mice. These effects are in line with the effects of haloperidol on stressed D1CT-7 mice^13^.

Several limitations of the study should be recognized. First, the translational value of these findings is partially limited by the evaluation of AP’s effects only in one model of TS. Nevertheless, it is worth noting that, among the currently available animal models of TS^10^, D1CT-7 mice feature unique characteristics with respect to face and predictive validity. For example, tic-like alterations in D1CT-7 mice are sex-dimorphic; furthermore, these responses are sensitive to all major TS therapies, such as haloperidol and clonidine^11, 13^. While the construct validity of D1CT-7 mice with respect to TS was not apparent at the time of their development, their brain pattern of neuropotentiation was found to be restricted to the somatosensory/ insular cortex and amygdala^11, 12^; notably, all these regions, and in particular the insular cortex, have been recently shown to be particularly relevant in the regulation of premonitory sensory phenomena^9, 27, 45^. Nevertheless, D1CT-7 mice feature phenotypes that may not be directly related to TS, such as their hyperactivity; thus, future studies in other models of TS are necessary to confirm the role of AP in stress-elicited exacerbation of symptoms. Second, we have shown that the doses of finasteride used in our experiments can reduce locomotor activity in mice; this observation raises the possibility that the effects of finasteride may be due to non-specific sedative effects. While it is possible that the hypolocomotion induced by this drug may have contributed to some of the effects reported in this study, this possibility is substantially tempered by the finding that the doses that reduced tic-like behaviors in D1CT-7 mice did not lead to a suppression of startle responses, a common feature of sedative drugs^46^. Third, although our results point to a key role of AP in the regulation of TS-like responses in D1CT-7 mice, we cannot exclude that other neurosteroids may also participate in the behavioral effects of stress. Further investigations will need to focus on other neurosteroids increased by stress such as tetrahydrodeoxycorticosterone or androgenic neuroactive steroids, as well as the role of the GABA-A receptor and other AP-sensitive receptors. These analyses may prove essential to help clarify the male predominance of TS, as well as potential mechanisms of comorbidity with other neuropsychiatric problems, including ADHD and OCD. Fourth, our analyses were only limited to the PFC of adult mice; however, it is likely that the effects of other neurosteroids may differ with age; furthermore, other regions, such as the nucleus accumbens, may be involved in the effects of finasteride^47^. Given the limitations in the size of this region, however, further improvements in our ability to detect neurosteroid levels will be needed to address this issue.

Despite these limitations and caveats, the present findings are the first to suggest the potential involvement of AP in the adverse effects of acute stress on tics and related sensory correlates. Future studies will be essential to confirm these findings in TS patients and explore the therapeutic potential of neurosteroid-targeting therapies in tic disorders.

## Methods

### Animals

We used 3–4-month-old, experimentally naïve male Balb/c mice (n = 8–15 per genotype and treatment group) weighing 20–30 g. Animals were purchased by Jackson Labs (Bar Harbor, ME) and genotyped as previously described^11^. Since the inheritance pattern of D1CT-7 mutation is autosomal dominant, we bred WT females with heterozygous D1CT-7 sires. This breeding scheme was selected to standardize maternal behavior. Animals were housed in group cages with *ad libitum* access to food and water. The room was maintained at 22 °C, on a 12 h: 12 h light/dark cycle from 8 am to 8 pm. Animals were tested during their light cycle between 12 and 4 pm to minimize any potential circadian effects. All experimental procedures were in accordance with the NIH guidelines and approved by the IACUCs of the Universities of Kansas and Utah.

### Drugs

The following drugs were used: progesterone, DHP, AP (Tocris Bioscience, Bristol, UK), finasteride (Astatech, Bristol, PA) and haloperidol (Sigma-Aldrich, Saint Louis, MO). Finasteride, progesterone, DHP and AP were suspended in 5% Tween 80, diluted with 0.9% saline, and administered by IP injection in a 10 ml/kg volume. Haloperidol was dissolved in 10% acetic acid buffered with NaOH and diluted with saline.

### Dissection of brain regions

Immediately after decapitation, brains were frozen and the frontal portion cut into 1-mm-thick slices using a Jacobovitz brain slicer (Zivic Miller, Portersville, PA). The slices obtained from 1.18 to 0.14 anterior to bregma were mounted on a coverslip kept at 4 °C and disks (1.5-mm diameter) were punched out from these slices.

### Neurochemical analyses

Extraction, derivatization, and GC-MS analyses of neurosteroids were performed with minor modifications as described^48, 49^. The steroid measurements included progesterone, DHP, AP, and pregnanolone (3α,5β-tetrahydroprogesterone). Expression of GABA-A receptor subunits (α1, α4, δ and π) was measured by western blot with a variant of the protocol described by Kralic and colleagues^50^. Full details are described in Supplementary Information.

### Behavioral Studies

Tic-like manifestations were scored by trained observers blinded to the treatment, as previously indicated^12^. Tic-like manifestations were defined as rapid (<1 second) twitches of the head and/or body. SC, PPI and locomotor analyses were carried out as previously described^13, 29^ (see Supplementary Information for full details).

### Statistical analyses

Normality and homoscedasticity of data distribution were verified by using Kolmogorov-Smirnov and Bartlett’s tests. Statistical analyses of parametric data were performed with one-way or multi-way ANOVAs, followed by Newman-Keuls’ test for *post-hoc* comparisons. The significance threshold was set at 0.05.

## Electronic supplementary material

Supplementary Information
