# Supplementary material for: Allopregnanolone mediates the exacerbation of Tourette-like responses by acute stress in mouse models
Source: Sci Rep. 2017 Jun 13;7:3348. doi: 10.1038/s41598-017-03649-1 (PMC5469807; doi:10.1038/s41598-017-03649-1)
Supplement: Supplementary file 1 — Supplementary Information [file 41598_2017_3649_MOESM1_ESM.pdf]

Allopregnanolone mediates the exacerbation of Tourette-like responses by acute stress in mouse models

Laura J Mosher, Sean C Godar, Marianela Nelson,

Stephen C Fowler, Graziano Pinna, Marco Bortolato

SUPPLEMENTARY METHODS

Measurement of neurosteroid content. Extraction, derivatization, and GC-MS analyses of neurosteroids were performed with minor modifications as described [1,2]. The steroid measurements included progesterone, 5α-dihydroprogesterone, allopregnanolone, and pregnanolone (3α, 5β-tetrahydro-progesterone). Supernatants were extracted with ethyl acetate and, after lyophilization, neurosteroids were purified and separated by HPLC, as previously described [2]. Tritiated neurosteroids (American Radiolabeled Chemicals, St. Louis, MO) were added to monitor retention time through HPLC [2,3] while deuterated internal standards (CDN Isotopes, Pointe-Claire, QC, and Steraloids, Newport, RI) were added to allow quantification of the compound of interest. The HPLC fractions containing progesterone, allopregnanolone, and pregnanolone were derivatized with heptafluorobutyric acid anhydride (HFBA) (Supelco, Bellefonte, PA). 5α-dihydroprogesterone was derivatized with N-methyl-N-(trimethylsilyl) trifluoroacetamide (MSTFA)-ammonium iodide (NH_4I)/1,4-dithioerythritol(DTE)/ acetonitrile(CH_3CN) (Sigma-Aldrich) in a ratio of 1,000/2/5/1,000, and subjected to GC-MS. Mass fragmentography analysis of derivatized hormones was performed in the standard electron impact mode with a detection limit of ≈10 fmol and intra-assay coefficients of variation less than 5%. Neurosteroids were identified based on their GC/MS retention time characteristics; the definitive structural identification of each neurosteroid was provided by its unique mass fragmentation pattern. To calculate the quantity of the neurosteroid of interest in each fraction, the area under the peak of the neurosteroid in the sample was divided by the area under the peak of the deuterated internal standard. Only peaks with a signal-to-noise ratio greater or equal to 5:1 were integrated.

Western Blot. Samples were homogenized on ice in a buffer containing 10 mM Tris-HCl pH 7.4, 5 mM EDTA, 320 mM sucrose, protease and phosphatase inhibitor cocktail. Homogenates were centrifuged for 5 min at 3000 x g to precipitate nuclei; supernatant fractions were collected and centrifuged at 20,000 x g for 60 min. The resulting pellet (P2) was solubilized in T-PER lysis buffer (Tissue Protein Extraction Reagent, Pierce, Rockford, IL) supplemented with protease and phosphatase inhibitor cocktail. Small

aliquots of the homogenate were used for protein determination by a modified Lowry protein assay method (DC protein assay, Bio-Rad Laboratories, Hercules, CA). Equal amounts of proteins were separated on a 4–15% Criterion TGX Precast Gel (Bio-Rad Laboratories) by electrophoresis and transferred to a polyvinylidene difluoride membrane using the Trans-Blot Turbo Transfer system (Bio-Rad Laboratories). Membranes were then blocked with 3% BSA (Sigma-Aldrich) in TRIS-buffered saline supplemented with 0.1% Tween 20 for 2 h at room temperature and then incubated overnight with primary antibodies (1:1000) at 4 °C. Primary antibodies used in this study include the following: anti-actin (ab3280, Abcam, Cambridge, MA); anti-GABA-A receptor α1 (NB 300-191, Novus Biologicals, Littleton, CO); anti-GABA-A receptor α4 (NB 300-194, Novus Biologicals); anti-GABA-A receptor δ (ab111048, Abcam); and anti-GABA-A receptor π (ab26055, abcam). These GABA-A receptor subunits were chosen as they express a high affinity for neurosteroids and have been indicated to regulate the binding of allopregnanolone and neurosteroids to GABA-A receptors [4-6]. After washing, membranes were incubated with HRP-conjugated secondary antibodies. Antibody binding was detected using Clarity ECL substrate (Bio-Rad Laboratories) and proteins were analyzed by the ChemiDoc Touch system and the Image Lab software (Bio-Rad Laboratories). Membranes were stripped and re-probed with an anti-actin antibody for normalization.

Spatial Confinement. Mice were confined for 20 min within a clear, bottomless Plexiglas cylinder (10 cm in diameter x 30 cm in height) in their home cage. Non-confined controls remained in their home cages. In order to avoid potential carry-over effects of spatial confinement stress, each animal was used only once in our experimental design.

Acoustic startle reflex and prepulse inhibition. The apparatus used for detection of startle reflexes (SR-LAB; San Diego Instruments, San Diego, CA) consisted of five Plexiglas cages (diameter: 5 cm) in sound-attenuated chambers with fan ventilation. Each cage was mounted on a piezoelectric accelerometric platform connected to an analogue-digital converter. The response to each stimulus was recorded as 65 consecutive 1-ms readings. A dynamic calibration system was used to ensure comparable sensitivities across chambers. The startle testing protocol featured a 70-dB

background white noise, and consisted of a 5-min acclimatization period, followed by three consecutive blocks of pulse, prepulse+pulse and “no-stimulus” trials. During the first and the third block, mice received only five pulse-alone trials of 115 dB. Conversely, in the second block mice were exposed to a pseudorandom sequence of 50 trials, consisting of 12 pulse-alone trials, 30 trials of pulse preceded by 73, 76 or 82 dB pre-pulses intensities (ten for each level of prepulse loudness) and eight no-stimulus trials, where only background noise was delivered. Intertrial intervals were selected randomly between 10 and 15 s. Sound levels were assessed using an A Scale setting. Percent prepulse inhibition was calculated with the following formula:

$$100 - \left(\frac{mean\ startle\ amplitude\ for\ prepulse\ pulse\ trials}{mean\ startle\ amplitude\ for\ pulse\ alone\ trials}\right) \times 100$$

The first and last blocks of 5 pulse-alone bursts were excluded from the calculation. As no interaction between prepulse levels and treatment were found in the statistical analysis, % prepulse inhibition values were collapsed across prepulse intensity to represent average % prepulse inhibition.

Analysis of locomotor behaviors. Spontaneous locomotor responses to novel environments were tested for 20-30 min in a square force plate actometer (side: 28 cm; height: 30 cm). Each of the four force plate actometers consisted of 4 force transducers placed at the corners of each loading plate. Transducers were sampled 100 times/s, yielding a 0.01 s temporal resolution, a 0.2 g force resolution, and a 2 mm spatial resolution. Custom software directed the timing and data-logging processes via a USB-1208FS interface (Measurement Computing, Norton, MA). Additional algorithms were used to extract macrobehavioral variables. Distance traveled, rotation bias, velocity, thigmotaxis, stride length and stride rate were all analyzed as previously described [7]. Distance traveled was calculated as the sum of the distances between coordinates of the location of the center of force recorded every 0.50 s over the recording session. Rotation bias was calculated by summing the locomotor turn direction over time using the center of the actometer floor as a reference point. Velocity was defined as the distance coved by a run divided by the duration of that run and expressed as mm/s (equivalent to the product of stride length and stride rate). Distance to the nearest wall

was used as an index of thigmotaxis. Stride length was obtained by dividing the distance covered by a run by the number of strides in that run for each subject. Stride rate was calculated for each trot by dividing the number of half strides by 2 to obtain the number of strides and then dividing the number of strides by the duration of the trot to yield stride rate expressed in Hz.

SUPPLEMENTARY REFERENCES

1. Pinna, G., Dong, E., Matsumoto, K., Costa, E. & Guidotti, A. In socially isolated mice, the reversal of brain allopregnanolone down-regulation mediates the anti-aggressive action of fluoxetine. *Proc. Natl. Acad. Sci. U. S. A.* **100**, 2035-2040 (2003).
2. Pinna, G. *et al.* Brain allopregnanolone regulates the potency of the GABA(A) receptor agonist muscimol. *Neuropharmacology* **39**, 440-448 (2000).
3. Rasmusson, A. M. *et al.* Decreased cerebrospinal fluid allopregnanolone levels in women with posttraumatic stress disorder. *Biol. Psychiatry* **60**, 704-713 (2006).
4. Bianchi, M. T., Haas, K. F. & Macdonald, R. L. Alpha1 and alpha6 subunits specify distinct desensitization, deactivation and neurosteroid modulation of GABA(A) receptors containing the delta subunit. *Neuropharmacology* **43**, 492-502 (2002).
5. Neelands, T. R. & Macdonald, R. L. Incorporation of the pi subunit into functional gamma-aminobutyric Acid(A) receptors. *Mol. Pharmacol.* **56**, 598-610 (1999).
6. Smith, S. S., Shen, H., Gong, Q. H. & Zhou, X. Neurosteroid regulation of GABA(A) receptors: Focus on the alpha4 and delta subunits. *Pharmacol. Ther.* **116**, 58-76 (2007).
7. Fowler, S. C., Mosher, L. J., Godar, S. C. & Bortolato, M. Assessment of gait and sensorimotor deficits in the D1CT-7 mouse model of Tourette syndrome. *J. Neurosci. Methods* (2017).

SUPPLEMENTARY TABLE 1.

NC WT	4.77 ± 4.12
NC D1CT7	0.7 ± 0.18
SC WT	2.38 ± 1.13
SC D1CT7	1.5 ± 0.34

Table S1. Levels of pregnanolone in the prefrontal cortex of D1CT-7 and wild-type (WT) littermates following spatial confinement (SC). Data are shown as means ± SEM (in pg/mg). NC, non-confined. N=4-7/group.

SUPPLEMENTARY FIGURE 1

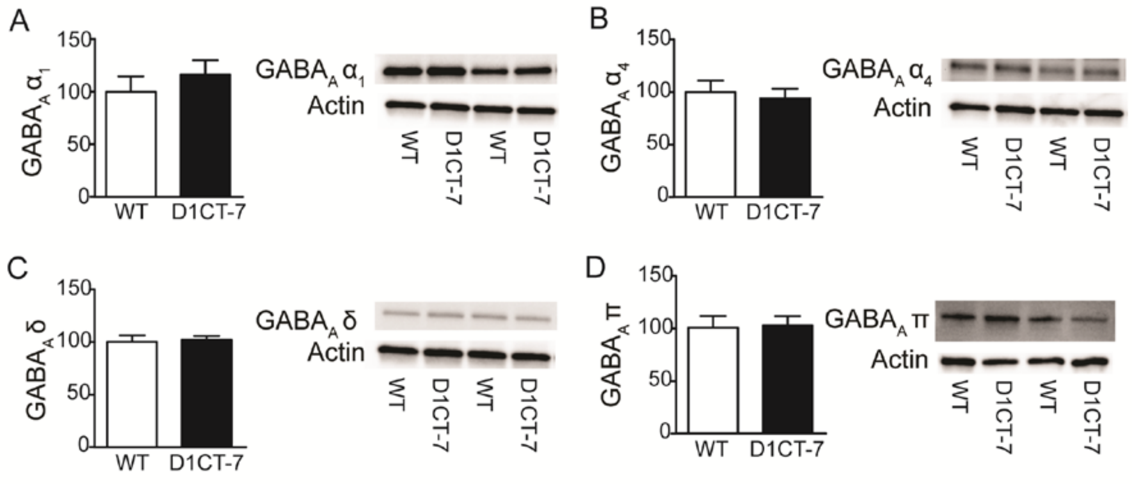

Fig. S1. Expression of GABA-A receptor (A) α_1, (B) α_4, (C) δ and (D) π subunits in the prefrontal cortex of D1CT-7 and wild type (WT) mice. Data are shown as means ± SEM. Representative gels for each subunit and actin controls are presented. Gel images have been cropped to the band of interest for each protein. N=6/group. For further details, see text.

SUPPLEMENTARY FIGURE 2

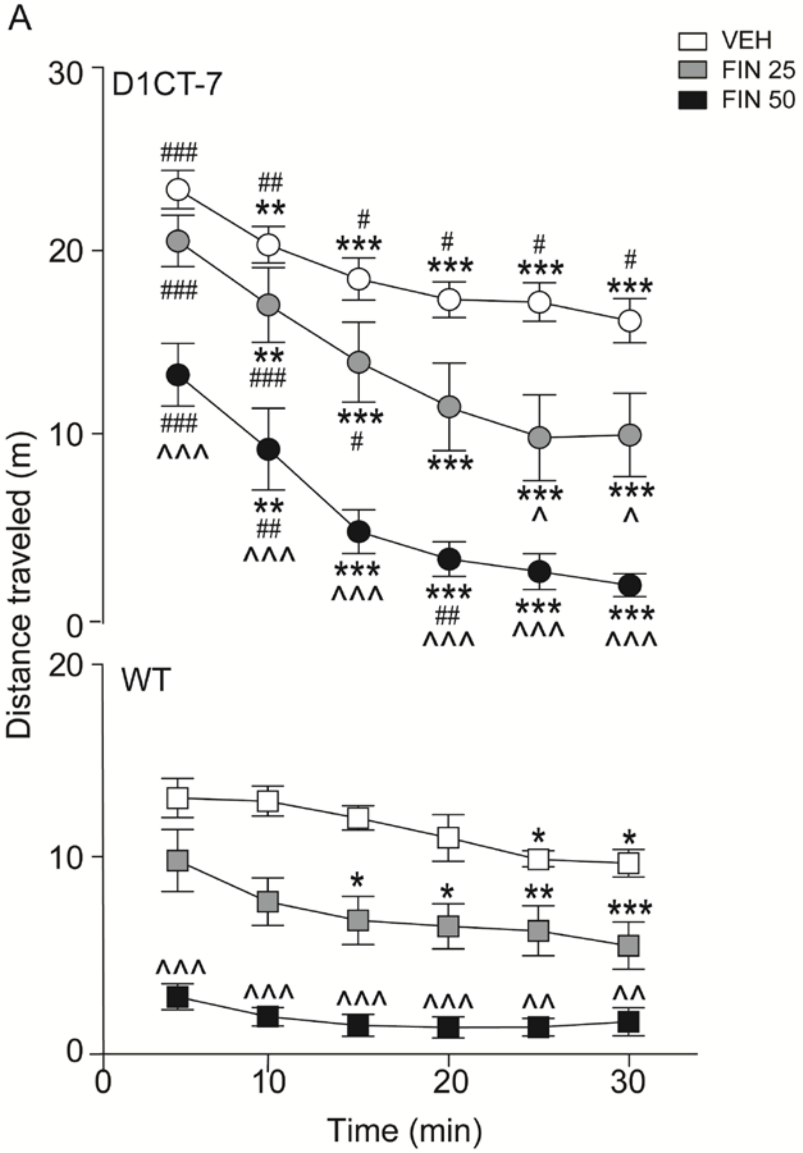

Fig. S2. Effects of finasteride (FIN; 25-50 mg/kg, IP) on locomotor activity of D1CT-7 and wild-type (WT) littermates. Data are shown as means ± SEM. $^{\#}P < 0.05$, $^{\#\#}P < 0.01$ $^{\#\#\#}P < 0.001$ for WT vs D1CT-7 of the same time block and treatment. $^{*}P < 0.05$, $^{**}P < 0.01$ and $^{***}P < 0.001$ for indicated time point vs the 1st time block of the same genotype and treatment. $^{\wedge}P < 0.05$, $^{\wedge\wedge}P < 0.01$ and $^{\wedge\wedge\wedge}P < 0.001$ for comparisons between the indicated time point vs VEH treated of the same genotype and time block. N = 8/group. Abbreviations: VEH, vehicle.
